# A robust fixed point transformation-based approach for type 1 diabetes control

**DOI:** 10.1007/s11071-017-3598-7

**Published:** 2017-06-19

**Authors:** Levente Kovács

**Affiliations:** Physiological Controls Research Center, Research and Innovation Center of the Óbuda University, Kiscelli Street 82., Budapest, 1032 Hungary

**Keywords:** Adaptive control, Diabetes control, Robust fixed point method, T1DM, RFPT

## Abstract

Modeling and control of diabetes mellitus (DM) are difficult due to the highly nonlinear attitude, time-delay effects, the impulse kind input signals and the lack of continuously available blood glucose (BG) level to be regulated. Regarding the mentioned problems, identification of DM model is crucial. Furthermore, due to the lack of information about the internal states (which cannot be measured in everyday life) and because the BG level is not available in every moment over time, adaptive robust control design method regardless exact model dependency would successfully handle these unfavorable effects without simplifications. The recently developed nonlinear robust fixed point transformation (RFPT)-based controller design method requires only a roughly approximate model in order to realize the controller structure. Moreover, parallel simulated approximate models—in order to provide additional internal information—can be used with the method. In this paper, the usability of the novel RFPT-based technique is demonstrated on the physiological problem of diabetes.

## Introduction

The concept of modern control technology, under the name “Theory of Governors,” originates from a paper by Maxwell [25], in which, without entering into any details of the particular mechanisms that were known at that time, he directed the attention of engineers and mathematicians to a more general dynamical theory. Following his fundamental achievements in the description of the electrical and magnetic phenomena [26], by the use of the elementary circuit components as resistors, capacitors and inductances as linear time-invariant (LTI) elements, in the field of electrical engineering rapid development was produced that intensively utilized the mathematical achievements of the nineteenth century as the analysis of complex numbers, Laplace, Z, Fourier, Mellin and other transforms [6]. In this great flourishment of linear control technology, the use of, and thinking on the basis of the frequency picture became prevailing. This general attitude lasted till the beginning of 1960s when, according to [19], Rudolf Kalman “...challenged the accepted approach to control theory of that period, limited to the use of Laplace transforms and the frequency domain, by showing that the basic control problems could be studied effectively through the notion of the state of the system that evolves in time according to ordinary differential equations in which control appears as parameters. ...Liberated from the confines of the frequency domain and further inspired by the development of computers, automatic control theory became the subject matter of a new science called systems theory.”

This liberation from the LTI systems tailored frequency domain-based problem tackling that opened the way for studying nonlinear dynamical systems in “system theory,” happened relatively lately in comparison with other revelations of fundamental significance in life sciences and chemistry, regarding biophysical and/or biochemical systems.

Lapicque [22] elaborated a strongly nonlinear model for describing neuron spiking. Hodgkin and Huxley [15] quantitatively modeled the membrane current in nerve excitation. In the early 1960s, further research results were published for the pulse transmission and membrane models [10, 27]. To ease the study of these nonlinear dynamic phenomena, L. Chua and T. Matsumoto constructed and studied a special nonlinear, but relatively simple electrical circuit [24]. In the last decade of the twentieth century, the significance of chaotic phenomena in the nervous system obtained general interest among the researchers [12, 29]. At the beginning of the twenty-first century, systematic, geometric and mathematical modeling of these phenomena was initiated [7, 13, 18], and the subject area of chaos synchronization obtained great attention as well [36, 40]. Nowadays, the combination of nonlinearity and fractional order dynamics became an interesting research area [2, 38].

In general, in biomedical problems the main sources of nonlinearities and variable coupling originate:

- From the mass action law due to which the products of various integer or rational powers of concentrations occur in the balance equations,
- Nonlinear truncations, because the physical interpretation does not allow negative concentrations,
- The limitation of the control signals because a reagent can have only positive ingress rate—it cannot be purely extracted from the stirring tank or the living organism,
- From the phenomena of “Input Coupling” [35], meaning that by adding some reagent to the system its other components are inevitably diluted.

Diabetes mellitus (DM) is a chronic disease of the human metabolic system regarding the malfunction in the production and utilization of insulin hormone. Several types of DM exist grouped on the reason of DM [1, 11]:

- The lack of insulin production classifies Type 1 DM (T1DM);
- Resistance against the effect of insulin categorizes Type 2 DM (T2DM);
- Both the aforementioned cases create Double DM (DDM);
- DM during pregnancy classifies Gestational DM (GDM);
- Finally, there are DM caused by genetic disorders.

The most dangerous type of DM is T1DM which occurs when the patients’ own immune system identifies the pancreatic $\beta$-cells—which producing the insulin hormone—as targets and destroys them during an autoimmune reaction. Because of the lack of internal insulin, the patients need external insulin in order to avoid metabolic collapse. Beside the short-term handling, maintaining the long-term variability of glycemia is also important to avoid the long-term side effects of the disease [5].

**Table 1:** Parameters of the model [23]

Name	Unit	Description
$k_\mathrm{l}$	mg/dL min	Internal glucose production by the liver
$k_\mathrm{b}$	mg/dL min	Glucose consumption by the brain
$k_\mathrm{si}$	mg/U min	Insulin-dependent glucose decrease rate
$k_\mathrm{r}$	min	Static gain constant of glucose
$k_\mathrm{u}$	min	Static gain constant of insulin
$T_\mathrm{u}$	min	Time constant of insulin dynamics
$T_\mathrm{r}$	min	Time constant of glucose dynamics
$V_\mathrm{i}$	dL	Insulin distribution volume
$V_\mathrm{B}$	dL	Blood volume
*M*	kg	Body weight

Diabetes is not curable, but treatable. The treatment depends on the type of diabetes; however, in case of T1DM and over time in T2DM this means external insulin administration by insulin pen (manual) or insulin pump (semiautomated) [17]. From engineering point of view, the best solution in order to reach a close-to-normal glycemia during DM treatment is the semiautomated insulin pump therapy, where the electromechanical device administers the required insulin based on a developed control algorithm [4].

Modeling and identifying DM models are not trivial tasks. Almost all available models contain high nonlinearities which make the control designing procedure difficult. The input time signals have impulse nature, since the meal intakes and the external insulin administration can be modeled as impulse functions [11, 31]. Moreover, the output of such models can be compared only with quantized blood glucose data, since the commercially available continuous glucose monitoring sensors (CGMS) [4] used with the insulin pump systems measure on every 5 min due to technological limitations.

In the recent years, several advanced control solutions appeared with regard to maintain the glycemia [30]. However, most of them are model-based solutions using different simplifications of the nonlinear problem because of the aforementioned unfavorable circumstances [16, 21].

The current research work focuses on a recently appeared robust nonlinear solution, the robust fixed point transformation (RFPT) control method [32], that does not need exact models just roughly approximation of the real-world problem. The RFPT-based design method is demonstrated on the DM control problem using two control design approaches based on the affine or non-affine model of the physiological problem.

The paper is structured as follows. The first section contains the detailed description of the applied diabetes model, while the second section introduces the RFPT-based methodology. In the third section, the controller design procedure is demonstrated followed by the research results. The final section contains the conclusions and future work possibilities.

## Diabetes model

In this study, a recently appeared glucose-insulin model is investigated, developed in order to increase the efficiency of identification from real patients’ samples [23]. The state-space representation of the model is given as follows:

1a $$\begin{aligned} \dot{G}(t) =&-k_\mathrm{si} I(t) + k_\mathrm{l} - k_\mathrm{b} + D(t) \end{aligned}$$

1b $$\begin{aligned} \ddot{I}(t) =&-\frac{1}{T^2_\mathrm{u}} I(t) - \frac{2}{T_\mathrm{u}} \dot{I}(t) + \frac{k_\mathrm{u}}{V_\mathrm{i} T^2_\mathrm{u}} u(t) \end{aligned}$$

1c $$\begin{aligned} \ddot{D}(t) =&-\frac{1}{T^2_\mathrm{r}} D(t) - \frac{2}{T_\mathrm{r}} \dot{D}(t) + \frac{k_\mathrm{r}}{V_\mathrm{B} T^2_\mathrm{r}} r(t) \end{aligned}$$

Table 1 contains the description of the model parameters , while there numerical values were taken from [23].

The model has two inputs, namely the external insulin infusion rate *u*(*t*) (U/h) and the carbohydrate (CHO) intake *r*(*t*) (mg/min$^2$), and one output, the glycemia, *G*(*t*) (mg/dL) used as a state of the model as well. Other states of the model are the insulinemia, *I*(*t*) (U/L) and the digestion of CHO, *D*(*t*) (mg/dL/min). The first subsystem (Eq. 1a) is responsible to simulate the glucose dynamics with regard to the external and internal glucose appearance, the effect of insulin and the internal insulin-independent glucose consumption. The second subsystem (Eq. 1b) describes the insulin dynamics including the changing of insulinemia and external insulin intake, while the third subsystem (Eq. 1c) presents the digestion dynamics and creates connection between the CHO *r*(*t*) in meal and *D*(*t*).

In order to determine the relative order of the necessary control, the order of the time-derivative of *G*(*t*) has to be found. This can be immediately set by the control signal *u*(*t*), the insulin ingress rate. For this, the “effect chain” of the control signal has to be clarified.

### Effect chain of the control signal

According to (1b), *u*(*t*) immediately influences the $\ddot{I}(t)$. Since $\ddot{I}(t)$ occurs in the third time-derivative of *G*(*t*), Eq. (1a) has to be differentiated two times:

2a $$\begin{aligned} \ddot{G}(t) =&-k_\mathrm{si} \dot{I}(t) + \dot{D}(t) \end{aligned}$$

2b $$\begin{aligned} \dddot{G}(t) =&-k_\mathrm{si} \ddot{I}(t) + \ddot{D}(t) \end{aligned}$$

Via substituting (1b) and (1c) into (2b), the control equation can be obtained:

3 $$\begin{aligned} \dddot{G}(t)= & {} \frac{k_\mathrm{si}}{T^2_\mathrm{u}} I(t) + \frac{2 k_\mathrm{si}}{T_\mathrm{u}} \dot{I}(t) - \frac{k_\mathrm{u} k_\mathrm{si}}{V_\mathrm{i} T^2_\mathrm{u}} u(t)\nonumber \\&- \frac{1}{T^2_\mathrm{r}} D(t) - \frac{2}{T_\mathrm{r}} \dot{D}(t) + \frac{k_\mathrm{r}}{V_\mathrm{B} T^2_\mathrm{r}} r(t) \end{aligned}$$

from which the necessary control signal *u*(*t*) for the prescribed $\dddot{G}(t)$ can be calculated:

4 $$\begin{aligned} u(t)= & {} -\frac{V_\mathrm{i} T_\mathrm{u}^2}{k_\mathrm{si} k_\mathrm{u}}\dddot{G}(t) + \frac{V_\mathrm{i}}{ k_\mathrm{u}} I(t) + \frac{2 V_\mathrm{i} T_\mathrm{u}}{ k_\mathrm{u}} \dot{I}(t)\nonumber \\&-\,\frac{V_\mathrm{i} T_\mathrm{u}^2}{k_\mathrm{si} k_\mathrm{u} T_\mathrm{r}^2} D(t)- \frac{2V_\mathrm{i} T_\mathrm{u}^2}{k_\mathrm{si} k_\mathrm{u} T_\mathrm{r}} \dot{D}(t)\nonumber \\&+\,\frac{V_\mathrm{i} T_\mathrm{u}^2 k_\mathrm{r}}{k_\mathrm{si} k_\mathrm{u} V_\mathrm{B} T_\mathrm{r}^2} r(t) \end{aligned}$$

From Eq. (4), it becomes clear that the *u*(*t*) control signal directly affects the $\dddot{G} (t)$—that means the control law has to cover this connection.

## The robust fixed point transformation-based adaptive control method

The idea of solving nonlinear equations via iterative techniques has long traditions in numerical computing. In a wider context, the original task can be transformed into a fixed point problem that in the next step can be solved by iteration. For instance, the Newton–Raphson algorithm is a classic example that seems to be one of the fundamental methodologies and attracts great attention even nowadays [8, 20, 28, 39]. In the sequel, the transformation of the adaptive control task into a fixed point problem is briefly highlighted. This is followed by the creation and the convergence properties of the iterative control signal.

### Determination of the relative order of the control task: the kinetic tracking error prescription and the “response function”

In order to determine the relative order of the control task, the first step is to consider the physical quantity for which a nominal time-variation or nominal trajectory $G^N(t)$ is defined in the given task.

This step can be made on the basis of purely kinetic considerations by trying to prescribe the appropriate order time-derivative of the controlled quantity that instantaneously can be affected by the control signal. As it will be seen in Sect. 4, in our case, according to the model in use, the third time-derivative of the glucose concentration of the blood $\dddot{G}$  (mg/dL min$^3$) can be directly influenced by the control input *u* (U/h) that is the external insulin infusion rate. For instance, by considering the integrated tracking error defined as $e_\mathrm{int}(t)\mathop {=}\limits ^{\text {def}}\int _{t_0}^t \left[ G^N(\xi )-G(\xi )\right] \mathrm{d} \xi$, and by introducing a positive real number $0<\Lambda \,(\hbox {s}^{-1})$, we may wish to have $\left( \frac{\mathrm{d}}{\mathrm{d}t}+\Lambda \right) ^4 e_\mathrm{int}(t) \equiv 0$ that yields the “desired system response” as:

5 $$\begin{aligned} \dddot{G}^\mathrm{Desired}(t)= & {} \dddot{G}^N(t)+4\Lambda \left( \ddot{G}^N(t)-\ddot{G}(t)\right) \nonumber \\&+\, 6\Lambda ^2 \left( \dot{G}^N(t)-\dot{G}(t)\right) \nonumber \\&+\, 4 \Lambda ^3 \left( G^N(t)-G(t)\right) +\Lambda ^4 e_\mathrm{int}(t)\nonumber \\ \end{aligned}$$

In the possession of an available approximate system model, in the given situation, the controller can estimate the appropriate value *u* that, if the model would be exact, just would generate this $\dddot{G}^\mathrm{Desired}(t)$ value. Due to modeling and/or state estimation errors, when this control signal *u* is applied on the actually controlled system, the realized (and measurable) “realized response,” i.e., $\dddot{G}(t)$ will differ from $\dddot{G}^\mathrm{Desired}(t)$. On this basis, a “response function” can be defined that for an arbitrary input $\dddot{G}^\mathrm{In}$ yields the realized response as $\dddot{G}=f\left( \dddot{G}^\mathrm{In},\ldots \right)$, in which in the place of the symbol “...” the zero-, first- and second-order derivatives of *G*(*t*) and the other state variables of the system can be understood. Since $\dddot{G}$ can be instantaneously modified by *u*, while the other arguments in the place of the symbol “...” vary only slowly, we can use the approximation $\dddot{G}\approx f\left( \dddot{G}^\mathrm{In}\right)$. In the lack of information on the exact model parameters, the analytical expression of *f* is not available for the controller. However, the pairs made of $\dddot{G}$ and $\dddot{G}^\mathrm{In}$ are always known: the input value is determined by the controller, and $\dddot{G}$ is measurable. In the sequel, by the use of the *response function*, an iteration is suggested to find the appropriate value $\dddot{G}_\star$ for which $\dddot{G}^\mathrm{Desired}=f\left( \dddot{G}_\star \right)$.

### Transformation of the control task into a fixed point problem: the “robust fixed point transformation”

Assume that we have a digital controller, and in each control step we can make exactly one step of iteration by the use of a function *H* defined as follows: $\dddot{G}^\mathrm{In}_{1}=\dddot{G}^\mathrm{Des}_{1}$, and

6 $$\begin{aligned} \dddot{G}^\mathrm{In}_{n+1}= & {} H\left( \dddot{G}^\mathrm{In}_{n},\dddot{G}^\mathrm{Des}_{n+1}\right) \nonumber \\&\mathop {=}\limits ^{\text {def}}\left( \dddot{G}^\mathrm{In}_{n}+K_\mathrm{c}\right) \cdot \left\{ 1+B_\mathrm{c} \left[ \tanh \left( A_\mathrm{c}\right. \right. \right. \nonumber \\&\left. \left. \left. \left[ f\left( \dddot{G}^\mathrm{In}_{n}\right) -\dddot{G}^\mathrm{Des}_{n+1}\right] \right) \right] \right\} -K_\mathrm{c} \end{aligned}$$

where the real numbers $K_\mathrm{c}$, $A_\mathrm{c}$, and $B_\mathrm{c}$ are the adaptive control parameters. This simple function was introduced in [33].

If $\dddot{G}^\mathrm{Des}_{n+1}$ varies slowly, the $\dddot{G}^\mathrm{Des} \approx \text{ constant }$ assumption can be done. Evidently, if $f\left( \dddot{G}_\star \right) =\dddot{G}^\mathrm{Des}$, Eq. (6) provides $\dddot{G}^\mathrm{In}_n = \dddot{G}_\star =\dddot{G}^\mathrm{In}_{n+1}$ that represents the solution of the control task, *the Fixed Point of function H*. The other trivial fixed point is $\dddot{G}^\mathrm{In}_n = -K_\mathrm{c} =\dddot{G}^\mathrm{In}_{n+1}$ that cannot be used for control purposes.

This construction evidently corresponds to the requirement of causality; the controller learns from the experience made in the “recent past”: the signal to be used in control cycle number $(n+1)$, i.e., $\dddot{G}^\mathrm{In}_{n+1}$, is created by the use of the signal in cycle *n*, i.e., $\dddot{G}^\mathrm{In}_{n}$, and by the observed response, i.e., $f\left( \dddot{G}^\mathrm{In}_{n}\right)$. In the next subsection, the convergence properties of the here defined sequence are considered.

### Guaranteeing the convergence of the iteration in general

As is well known, a Banach space (as a set, casually denoted by $\mathscr {B}$) by definition is a complete, linear, normed metric space [14], i.e., it has the following properties:

- Linearity: $\forall \alpha , \beta \in \mathbb {C}$ and $x,\,y \in \mathscr {B}$, the *linear combination* is defined and belongs to the space: $\alpha x + \beta y \in \mathscr {B}$;
- Existence of a norm for defining a metrics: $\forall x \in \mathscr {B}$ $\exists \Vert x\Vert \ge 0$ so that from $\Vert a\Vert =0$ it follows that $a=0$ (i.e., the norm separates points), $\forall \alpha \in \mathbb {C}$ $\Vert \alpha x\Vert =|\alpha |\cdot \Vert x\Vert$ (absolute scalability), and $\Vert x+y\Vert \le \Vert x\Vert +\Vert y\Vert$ (norm inequality); by the use of this norm, the *metrics* or the distance between the elements as $\rho (x,y) \mathop {=}\limits ^{\text {def}}\Vert x-y\Vert$ can be defined;
- Completeness: By the use of the concept of the norm the so-called Cauchy-sequences can be defined as follows: a sequence $\{x_n; n \in \mathbb {N}\}$ is a Cauchy-sequence if $\forall L \in \mathbb {N}$ $\Vert x_{n+L}-x_{n}\Vert \rightarrow 0$ as $n \rightarrow \infty$; completeness means that each Cauchy-sequence must be convergent in a complete space, i.e., for the above sequence $\exists x_\star \in \mathscr {B}$ so that $\Vert x_n-x_\star \Vert \rightarrow 0$ as $n \rightarrow \infty$.

By the use of the norm, for the functions $\varPhi : \mathscr {B} \mapsto \mathscr {B}$, contractive ones can be defined as follows: $\varPhi$ is contractive if $\forall x \in \mathscr {B}$ $\exists K \in [0,1)$ so that $\Vert \varPhi (x) - \varPhi (y)\Vert \le K \Vert x-y\Vert$. By the use of a contractive function, Cauchy-sequences can be generated in the following manner: $\{x_1;x_2 \mathop {=}\limits ^{\text {def}} \varPhi (x_1);\ldots x_{n+1} \mathop {=}\limits ^{\text {def}} \varPhi (x_n); \ldots \}$. This sequence is evidently a Cauchy-sequence since:

7 $$\begin{aligned} \Vert x_{n+L}-x_n\Vert= & {} \Vert \varPhi (x_{n-1+L})-\varPhi (x_{n-1})\Vert \le \nonumber \\\le & {} K \Vert x_{n-1+L}-x_{n-1}\Vert \le \ldots \nonumber \\\le & {} K^{n-1} \Vert x_{1+L}-x_{1}\Vert \rightarrow 0 \text{ as } n \rightarrow \infty \nonumber \\ \end{aligned}$$

Due to the completeness of $\mathscr {B}$, $\exists x_\star \in \mathscr {B}$ so that $\Vert x_n - x_\star \Vert \rightarrow 0$ as $n \rightarrow \infty$. It is easy to show that $x_\star$ is the *Fixed Point* of $\varPhi$, i.e., $\varPhi (x_\star )=x_\star$. By utilizing the properties of the norm, it can be written that:

8 $$\begin{aligned}&\Vert \varPhi (x_\star )-x_\star \Vert = \Vert \varPhi (x_\star )-x_n+x_n-x_\star \Vert \nonumber \\&\quad \le \Vert \varPhi (x_\star )-x_n\Vert + \Vert x_n-x_\star \Vert \nonumber \\&\quad =\Vert \varPhi (x_\star )-\varPhi (x_{n-1})\Vert + \Vert x_n-x_\star \Vert \nonumber \\&\quad \le K \Vert x_\star -x_{n-1}\Vert + \Vert x_n-x_\star \Vert \rightarrow 0 \text{ as } n \rightarrow \infty \nonumber \\ \end{aligned}$$

As a result, the advantage of Banach spaces is the use of the above simple and practical argumentation in the case of quite “abstract” and “complicated” sets [3]. For instance, the quadratically integrable functions of modern quantum mechanics form a Hilbert-Space, that is only a special example of Banach spaces.

The above simple considerations allowed the application of the fixed point transformation-based approach for the control of systems of $\mathbb {R}^n \mapsto \mathbb {R}^n,\, n \in \mathbb {N}$-type response functions and made it possible to further clarify the conditions of convergence in [9].

### Convergence conditions for SISO systems

In our case, the $\dddot{G} \approx f\left( \dddot{G}^\mathrm{In}\right)$ response function corresponds to $f: \mathbb {R} \mapsto \mathbb {R}$, so we can use the Banach space of real numbers with the norm $\Vert x\Vert \mathop {=}\limits ^{\text {def}} |x|$. Since our function is differentiable, we can use a simple integral estimation for guaranteeing contractivity: $|f(b)-f(a)|=\left| \int _a^b \frac{\mathrm{d}f(x)}{\mathrm{d}x} \mathrm{d}x \right| \le \int _a^b \left| \frac{\mathrm{d}f(x)}{\mathrm{d}x}\right| \mathrm{d}x$, therefore if it can be guaranteed that $\mathop {0 \le K <1}\limits ^{\exists }$ so that $\left| \frac{\mathrm{d}f(x)}{\mathrm{d}x}\right| \le K$, a contractive map can be obtained that makes the iterative sequence converge to the solution of the control task.

Consequently, it is enough to maintain the contractivity nearby the useful fixed point. It is easy to see that if $|K_\mathrm{c}|\gg |\dddot{G}|$, $B_\mathrm{c} = \pm 1$, and $A_\mathrm{c}$ is a small positive number, the not useful fixed point at $-K_\mathrm{c}$ can be made *repulsive*, while $\dddot{G}_\star$ can be made *attractive*. Since $|K_\mathrm{c}|$ is very big, in this case the *initial element of the iteration* will be either in the $(-K_\mathrm{c}, \,\dddot{G}_\star )$ interval or it will be greater than $\dddot{G}_\star$; therefore, the iteration will converge to $\dddot{G}_\star$. An advantage of this control method is that it does not require very precise setting of its adaptive control parameters. The actual setting concerns the speed of convergence, therefore, to some extent the precision of trajectory tracking. In the field of life sciences, this fact is very important from practical point of view, because the method is not based on “exact proofs” for which the necessary conditions rigorously have to be guaranteed.

## Controller design

In the followings, the controller design method is demonstrated in the given case by using the aforementioned theorems and methods started with the realization of the affine and approximate system models.

### The affine model

It is evident that (4) corresponds to an affine structure as the relationship between *u*(*t*) and $\dddot{G}(t)$ is concerned. It is reasonable to assume that any abrupt jump in *u*(*t*) immediately affects the instant value of $\dddot{G}(t)$; hence, the “additive” parts of the affine model vary only slowly. As a result, in the RFPT-based control design the actual value of the control sequence $r_n$ will be the “required” third derivative of *G*. It will be referred to as $\dddot{G}(t)_\mathrm{Req}$ in the sequel. According to the available model to $\dddot{G}(t)_\mathrm{Req}$, the control signal $u(t)_\mathrm{Req}$ becomes as follows:

9 $$\begin{aligned} u(t)_\mathrm{Req}= & {} -\frac{V_\mathrm{i} T_\mathrm{u}^2}{k_\mathrm{si} k_\mathrm{u}}{\dddot{G}}_\mathrm{req}(t) + \frac{V_\mathrm{i}}{ k_\mathrm{u}} I(t)\ + \frac{2 V_\mathrm{i} T_\mathrm{u}}{ k_\mathrm{u}} \dot{I}(t)\nonumber \\&-\,\frac{V_\mathrm{i} T_\mathrm{u}^2}{k_\mathrm{si} k_\mathrm{u} T_\mathrm{r}^2} D(t)- \frac{2 V_\mathrm{i} T_\mathrm{u}^2}{k_\mathrm{si} k_\mathrm{u} T_\mathrm{r}} \dot{D}(t) + \frac{V_\mathrm{i} T_\mathrm{u}^2 k_\mathrm{r}}{k_\mathrm{si} k_\mathrm{u} V_\mathrm{B} T_\mathrm{r}^2} r(t)\nonumber \\ \end{aligned}$$

The phenomenological restrictions that are so typical in the control of T1DM obtain significance at this point.

Practically only *G*(*t*) can be measured by an appropriate continuous glucose monitoring sensor with 5 min cycle time. No direct measurement possibilities exist for measuring *D*(*t*) and *I*(*t*) in the practice. However, in principle *r*(*t*) may be known, as it depends on the action of the patient, but it cannot be expected that the patient “manually” provides the controller with this information. Therefore, it is assumed that the controller can “detect” the CHO intake through observing some increase in *G*(*t*).

Consequently, it can be stated that in practice there is no viable way to obtain information on the actual value of the additive parts of the affine model. The main feature of the RFPT-based adaptive controller that can work with an incomplete model and approximate it well fits to this practical problem: we do not need the application of complicated state estimators to estimate this term. In our model, this unknown contribution is denoted as an “AffineAdditive” constant term as follows:

10 $$\begin{aligned} u(t)_\mathrm{Req} = -\frac{ V_\mathrm{i} T_\mathrm{u}^2}{ k_\mathrm{si} k_\mathrm{u}}\dddot{G}_\mathrm{req} + \text{ AffineAdditive } \end{aligned}$$

In this approach, the information on the variables *D*(*t*) and *I*(*t*) is completely neglected.

### Approximate model

As no real measurements can be done for the estimation of the actual *D*(*t*) and *I*(*t*) values of the patient, an alternative possibility is the application of some “approximate model” (its parameters are denoted by the symbol $\sim$) to estimate them by solving the complete equations of motion for the approximate model taking the same control signal as the actual patient *u*(*t*), and producing the drift of “approximate state variables” $\dot{\tilde{G}}(t)$, $\dot{\tilde{I}}(t)$, $\dot{\tilde{D}}(t)$. In the estimation of $u(t)_\mathrm{Req}$ the “approximate quantities” $\tilde{I}(t)$, $\dot{\tilde{I}}(t)$, $\tilde{D}(t)$, $\dot{\tilde{D}}(t)$ are substituted from a computer program that emulates the behavior of the approximate model, but it takes the measurable actual *G*(*t*) value and—in the lack of information—instead of the actual input *r*(*t*) it takes zero:

11 $$\begin{aligned} u(t)_\mathrm{Req}= & {} -\frac{\tilde{V}_\mathrm{i} \tilde{T}_\mathrm{u}^2}{\tilde{k}_\mathrm{si} \tilde{k}_\mathrm{u}}\dddot{G}_\mathrm{req} + \frac{\tilde{V}_\mathrm{i}}{\tilde{k}_\mathrm{u}} \tilde{I}(t) + \frac{2 \tilde{V}_\mathrm{i} \tilde{T}_\mathrm{u}}{\tilde{k}_\mathrm{u}} \dot{\tilde{I}}(t)\nonumber \\&-\,\frac{\tilde{V}_\mathrm{i} \tilde{T}_\mathrm{u}^2}{\tilde{k}_\mathrm{si} \tilde{k}_\mathrm{u} \tilde{T}_\mathrm{r}^2} \tilde{D}(t)- \frac{2\tilde{V}_\mathrm{i} \tilde{T}_\mathrm{u}^2}{\tilde{k}_\mathrm{si} \tilde{k}_\mathrm{u} \tilde{T}_\mathrm{r}} \dot{\tilde{D}}(t) + 0 . \end{aligned}$$

This approach may give less work to the adaptive compensation than that of the simple “affine model.”

### Control law

During the study, a kinematic-type PID-control law was used. This is an appropriate choice if the goal of the control is trajectory tracking as it is in this case. Since the control signal affects the third derivative of the variable to be regulated, the control law of the same order has to be used:

12 $$\begin{aligned} \bigg ( \frac{\mathrm{d}}{\mathrm{d}t}+\Lambda \bigg ) ^4 \int \limits _{t_0}^{t} \big (G^N(\xi )-G(\xi )\big ) \mathrm {d}\xi = 0 \end{aligned}$$

which determines the following desired $\dddot{G}^\mathrm{Desired}(t)$ function:

13 $$\begin{aligned}&\dddot{G}^\mathrm{Desired}(t)= \bigg (\frac{\mathrm{d}}{\mathrm{d}t}\bigg )^3 G^N(t) \nonumber \\&\quad +\sum _{s=0}^{3} \left( {\begin{array}{c}4\\ s\end{array}}\right) \Lambda ^{4-s} \bigg (\frac{\mathrm{d}}{\mathrm{d}t}\bigg )^s \int \limits _{t_0}^{t} \big (G^N(\xi )-G(\xi )\big ) \mathrm{d}\xi \nonumber \\ \end{aligned}$$

where the $G^N(t)$ is the reference (nominal) blood glucose (BG) level, *G*(*t*) is the actual (real) BG level, and the error is the $G^N(t)-G(t)$ that has to converge to zero over time.

### Final control environments

**Fig. 1:**
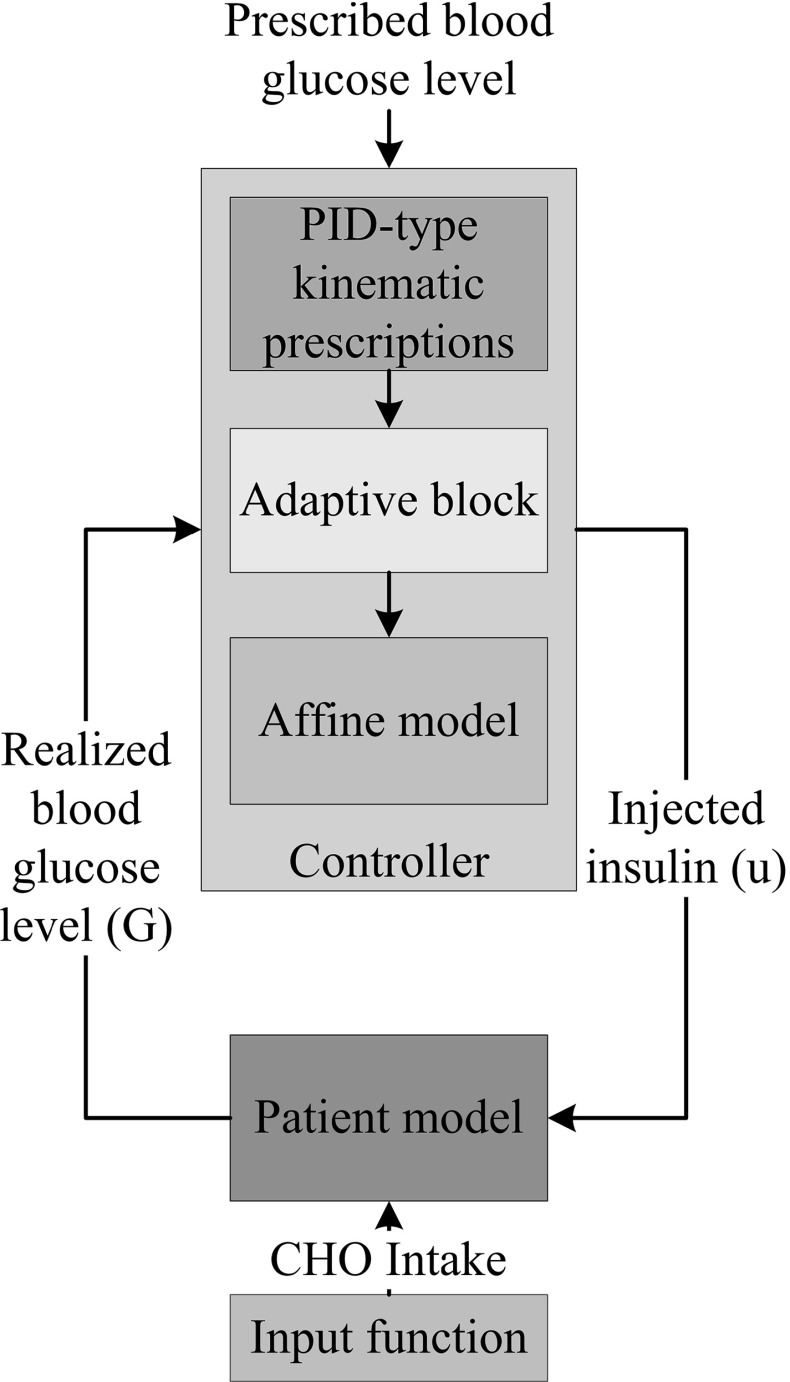
Control system realization in the affine case

**Fig. 2:**
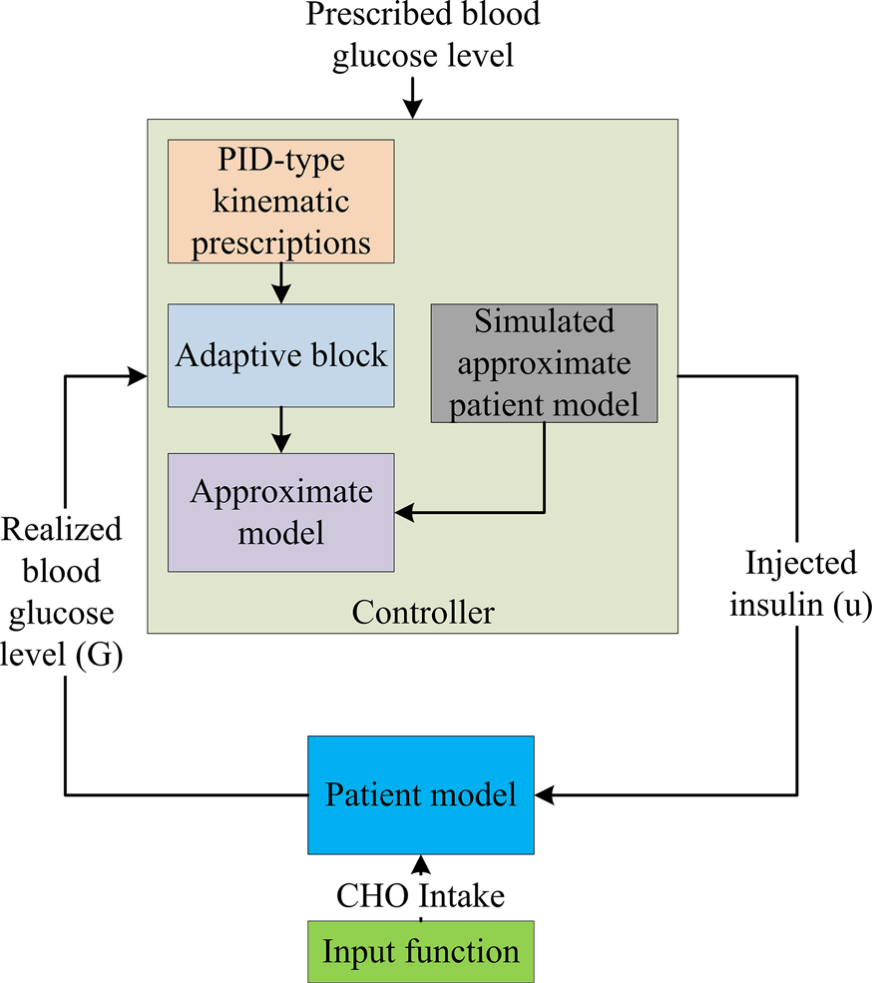
Control system realization in the non-affine case

The final control environment consists of the PID-type kinematic prescriptions (the control law), the adaptive block and the affine model (Fig. 1). However, in this study another realization possibility was investigated as well, where an approximate parallel simulated model provides the non-measurable estimated states. The structure of it is presented in Fig. 2. Note that the PID-type prescription and the adaptive block were the same.

## Results

In order to test the controller in the case of unfavorable circumstances, long-term simulations were applied and a unique glucose input function was designed. In every case, the goal was that the controller should provide an appropriate control signal by which the glycemia of a patient can be stable and appropriate in long-term beside continuous glucose disturbances.

**Fig. 3:**
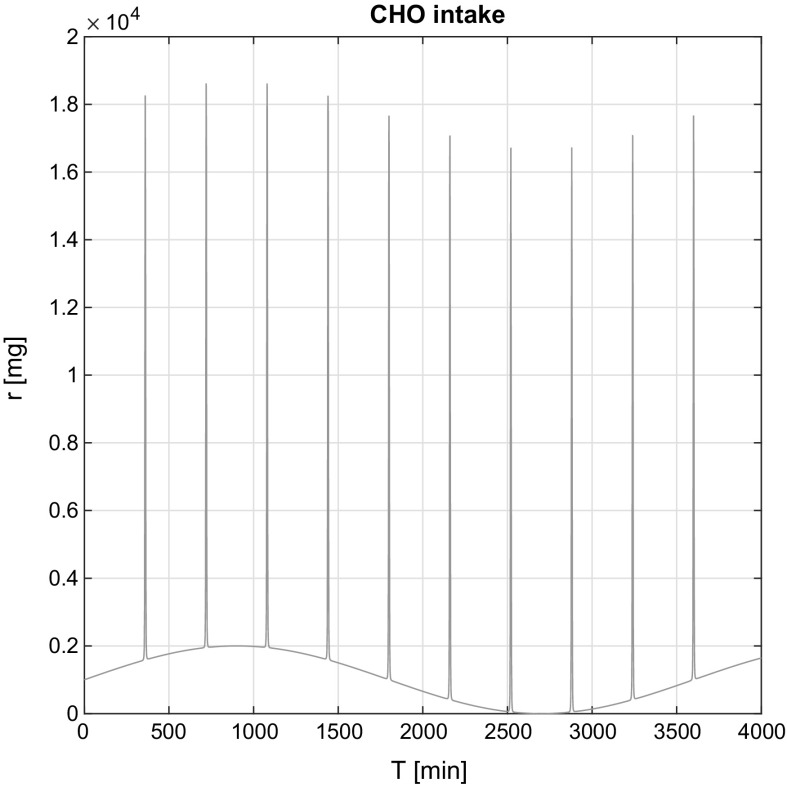
Used carbohydrate CHO intake

The glucose input specificity of the used model required a special input function. The designed function consists of an additive mixture of an arbitrarily selected sinusoidal disturbance signal and an impulse kind signal, *r*(*t*):

14 $$\begin{aligned} r(t) = \sin (t) + \frac{s_\mathrm{h}}{s_\mathrm{w}+(t-t_{i})^4)} \end{aligned}$$

where the $s_\mathrm{h}$ is the impulse height and $s_\mathrm{w}$ is the impulse width of the signal. The model needs the derivative of the designed function $\mathrm{d} r(t) /\mathrm{d}t$ as CHO input. Figure 3 shows the output of the designed specific CHO input function.

The primary goal of this study was to prove the usability of the RFPT-based controller design opportunity in case of the T1DM model created in order to ease the identification procedures. The method requests approximated models instead of exact patient models that allows using one of the parameter sets from the study of the model belonged to an identified patient (Patient 1, [23]) given in Table 2.

**Table 2:** Exact parameters of the used model [23]

Name	Unit	Value
$k_\mathrm{l}$	mg/dL min	1.94
$k_\mathrm{b}$	mg/dL min	128 / *M*
$k_\mathrm{si}$	mg/U min	197
$k_\mathrm{r}$	min	$2.4 V_\mathrm{B}\,10^{-3}$
$k_\mathrm{u}$	min	$59 V_\mathrm{i}\,10^{-3}$
$T_\mathrm{u}$	min	122
$T_\mathrm{r}$	min	183
$V_\mathrm{i}$	dL	2.5*M*
$V_\mathrm{B}$	dL	0.65*M*
*M*	kg	72

The AffineAdditive elements were set to zero during the simulation. Naturally, the parameters from Table 2 can only be used in the affine case. In the approximate case, the values of the state variables are non-measurable; however, roughly estimable from parallel simulation enough in order to efficiently use the RFPT-based method. The parameters in this case were half of the original values; namely, except the body weight, every parameter in the approximate case was equal with 0.5 times of its original value.

Beside the selection of the used model parameters, the appropriate selection of the control parameters is also important. The general RFPT-based controller parameters are those connected to the adaptivity, namely $A_\mathrm{c}$, $B_\mathrm{c}$ and $K_\mathrm{c}$. Moreover, depending on the applied control law, different further control parameters may occur. In this study, a kinematic control law was used (see Sect. 4.3) with one tunable $\Lambda$ gain parameter. The values of the adaptivity parameters were adjusted to the magnitudes of the controlled variable (the third derivative of *G*(*t*)). The last selectable variable is the reference (nominal) BG level $G^N$ which is used in the adaptivity block and the control law as well. Due to the desired goal, to prove the usability of the RFPT-based controller, the same control parameters and reference BG level were used during the simulations, without online parameter tuning. However, in other applications these properties of the RFPT-based controller design were successfully tested [34, 37]. The selected control variables of the T1DM case is given in Table 3.

**Table 3:** Selected control parameters of the T1DM casetable

Name	Value	Unit
$K_\mathrm{c}$	$-10^{-2}$	
$A_\mathrm{c}$	$1/(10 |K_\mathrm{c}|)$	
$B_\mathrm{c}$	1	
$G^N$	100	mg/dL
$\Lambda$	0.003	

The arbitrarily selected simulation length was 4000 min (more then 66 h, or almost 3 days), which is enough to demonstrate the benefit of the RFPT-based controller, i.e., the controller adapts to the patient’s needs.

**Fig. 4:**
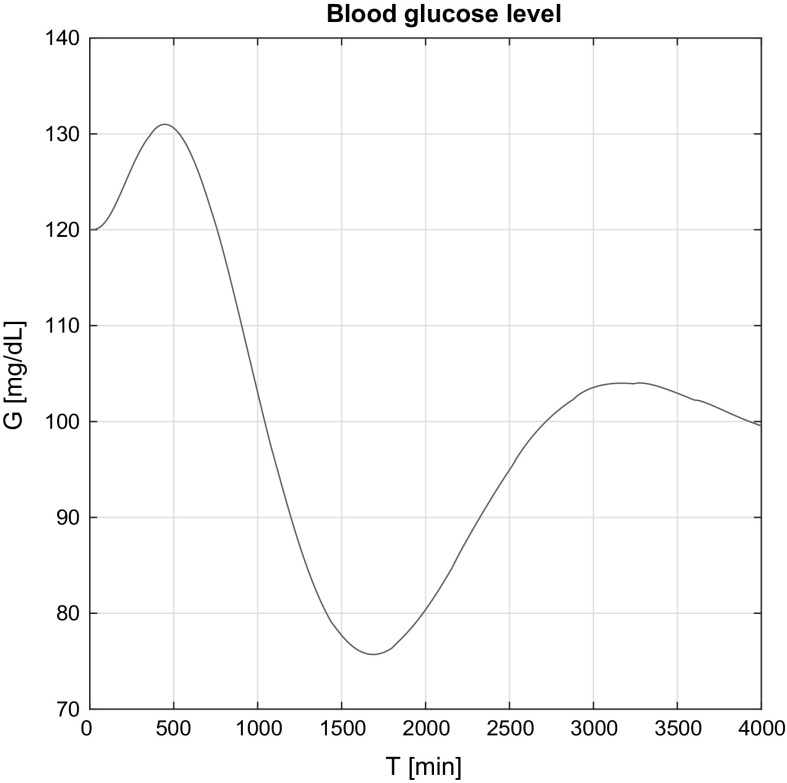
Simulation results of the blood glucose level in the affine case

Figure 4 presents the blood glucose level over time in the affine case. It should be noted that the desired blood glucose interval is 70–120 mg/dL (the healthy human blood glucose interval). Over 120 mg/dL hyperglycemia (high BG), under 70 mg/dL hypoglycemia (low BG) is diagnosed. The latter is the most dangerous for a T1DM patient and should be completely avoided. Hyperglycemia, however, could be tolerated, but the amplitude should be reduced to 140 mg/dL.

Evaluating the results presented in Fig. 4, it can be seen that after the first transient the controller reacts to the increasing BG level and administers insulin in order to avoid hypergycemia. As a result, the BG level finally reaches the nominal BG level $G^N$; however, the controller was continuously operating to prevent the unfavorable effects.

**Fig. 5:**
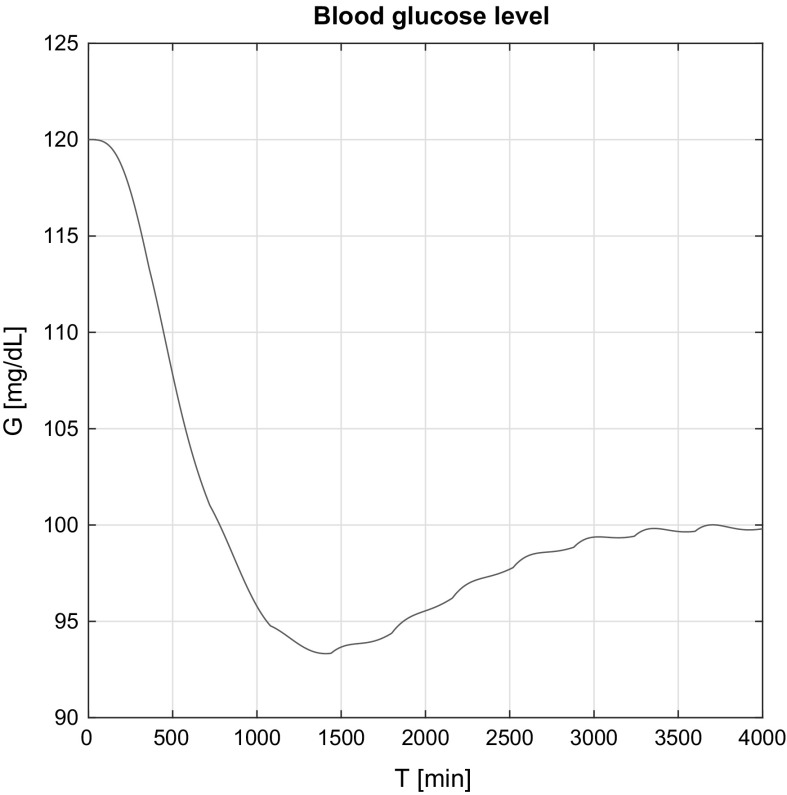
Simulation results of the blood glucose level in the non-affine case

In the non-affine case of Fig. 5, the controller acts faster, since the simulated approximate model signals are available and the controller has direct, but roughly approximated information about the possible internal states. The mild waviness in the figures is the effect of the control (insulin) signal, while the peaks occurred are discussed later.

**Fig. 6:**
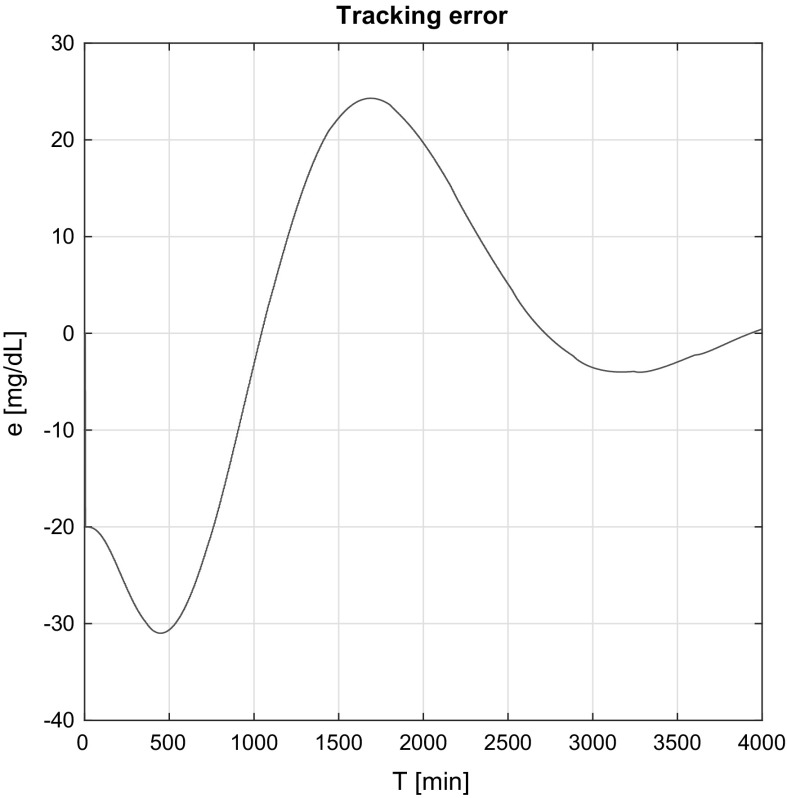
Tracking error simulation result in the affine case

**Fig. 7:**
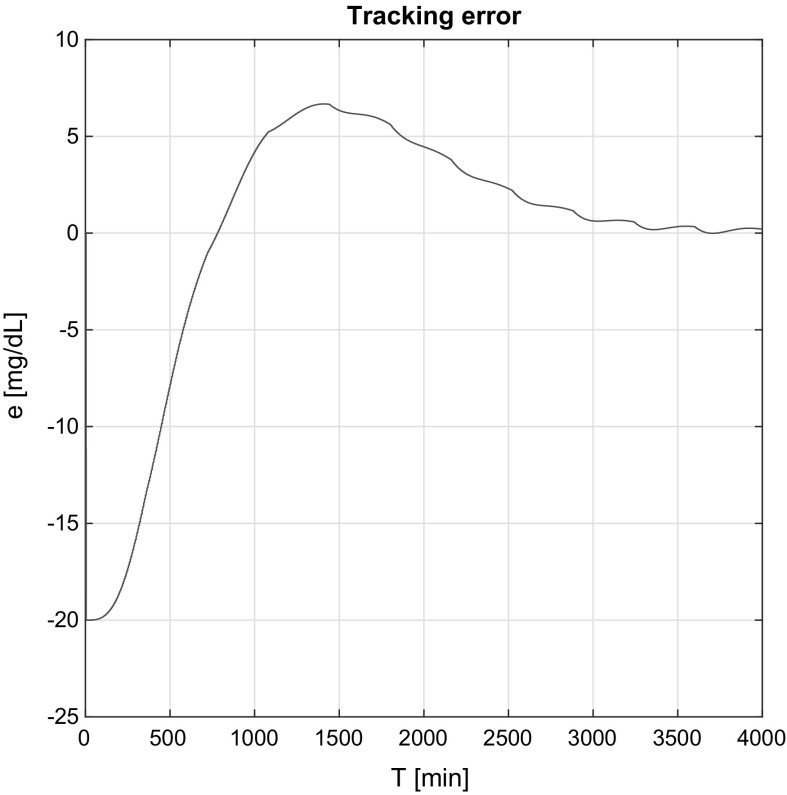
Tracking error simulation result in the non-affine case

Figures 6 and 7 show the tracking errors in the affine and non-affine cases. The same conclusions can be observed; namely, in the affine case the tracking error decay is slower (as the RFPT-based controller did not have only indirect information), but the controller works efficiently over time. The waviness effect appears here as well, since the error signal stands from $G^N-G_\mathrm{realized}(t)$.

**Fig. 8:**
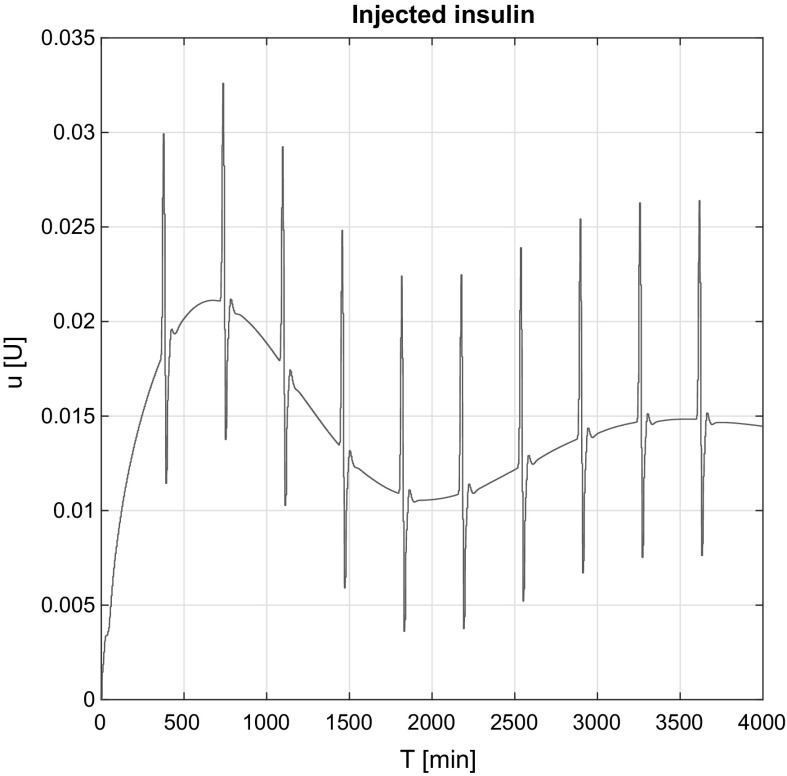
Injected insulin in the affine case

Figure 8 shows the injected insulin over time in the affine case. Due to the affine model’s structure (Eq. 9) insulin peaks occur over time because the third derivative of the required *G*(*t*). Originally, these effects come from the food intake signals and reflects in the $\dddot{G}(t)_\mathrm{Req}$ signal, respectively.

**Fig. 9:**
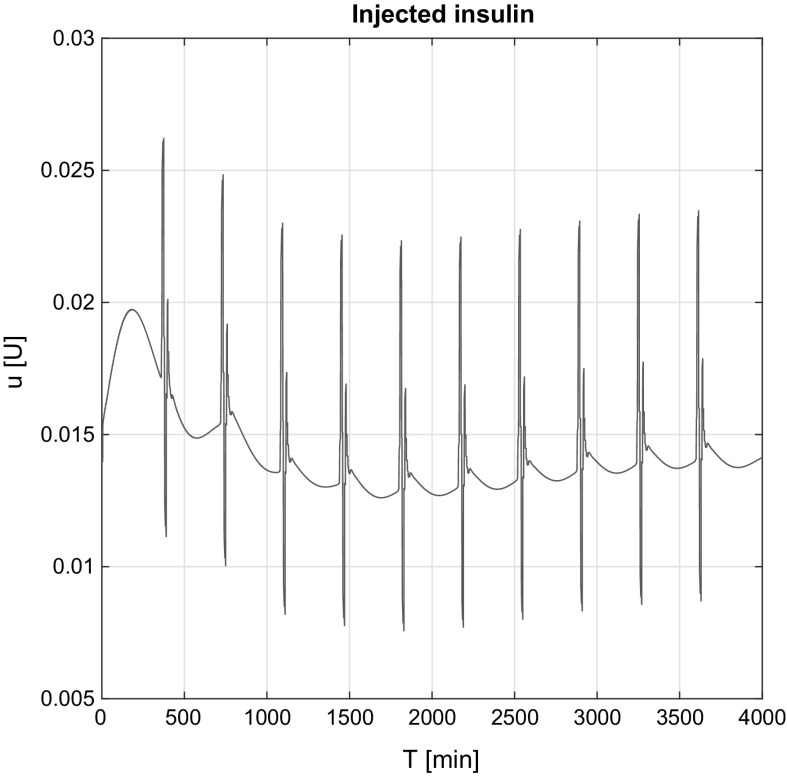
Injected insulin in the non-affine case

The same effects can be seen in the non-affine case (Fig. 9). The average magnitude of the peaks is almost the same. The main difference is that the controller has approximated internal information about the states. This knowledge insulinemia that allows us to have a nonzero initial control signal and have the non-smooth insulin signal around the peaks.

**Fig. 10:**
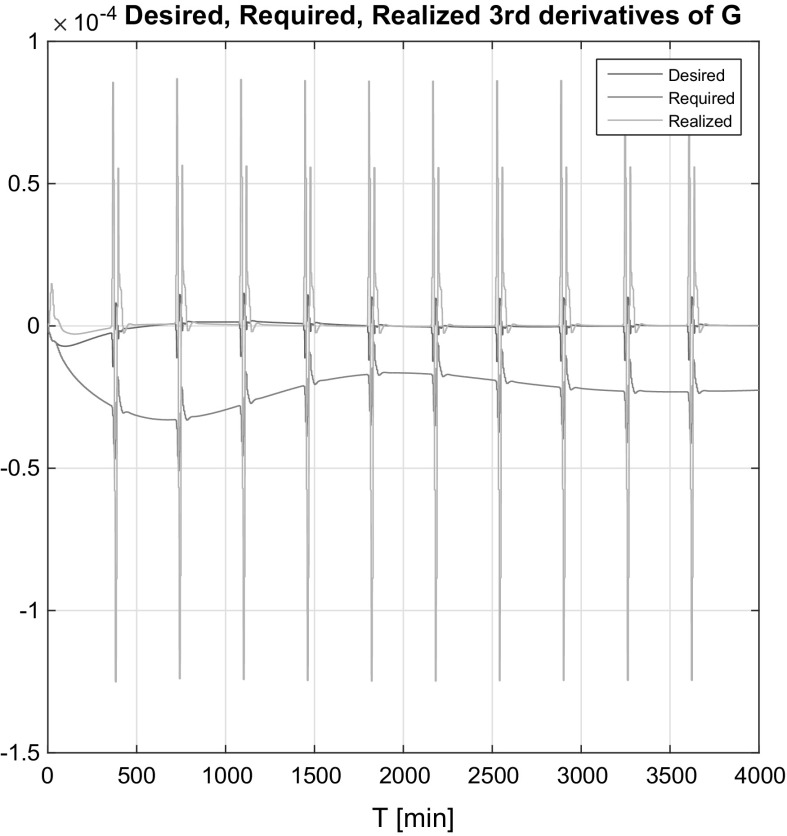
Desired, required, realized third derivatives of *G*(*t*) in the affine case

**Fig. 11:**
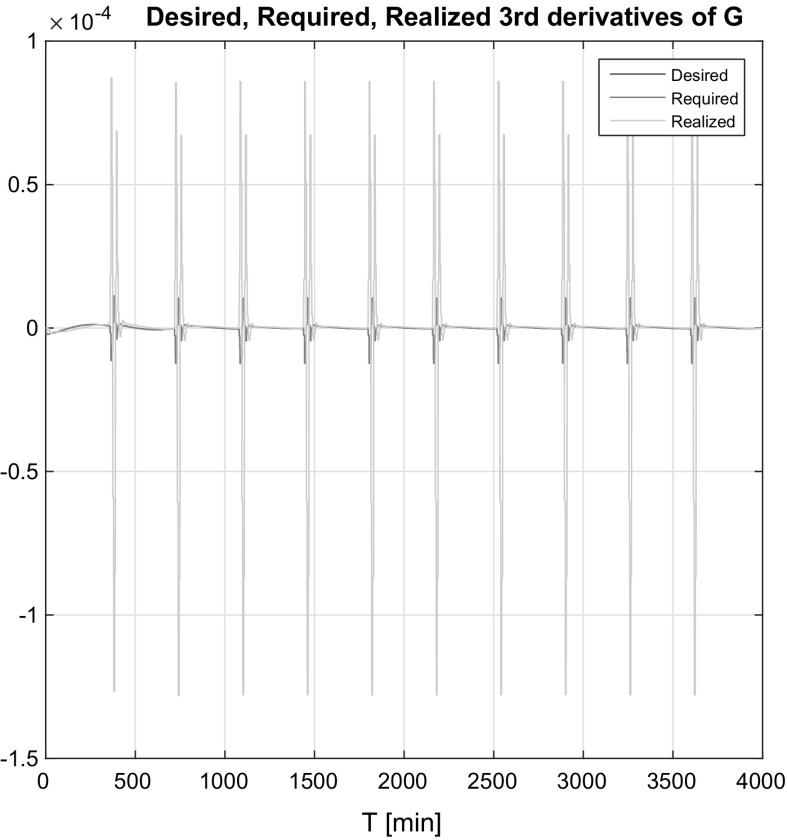
Desired, required, realized third derivatives of *G*(*t*) in the non-affine case

In Figs. 10 and 11, one can see the output of the realized (the real system’s) answer, desired (based on the control law) and required (recommendation of the adaptivity block) third derivatives of the BG level *G*(*t*) in both the affine and non-affine cases. In Fig. 10, the realized signal tends to the desired signal after the diversion caused by the insulin signals; hence, the adaptation works well. In Fig. 11 the desired and required signals are almost the same, which is the direct consequence of that fact that the controller has internal, however, roughly approximated information about the states. The realized $\dddot{G}(t)$ was tended to the desired signal as the low magnitudes and due to the fact that the desired and required signals were almost the same.

**Fig. 12:**
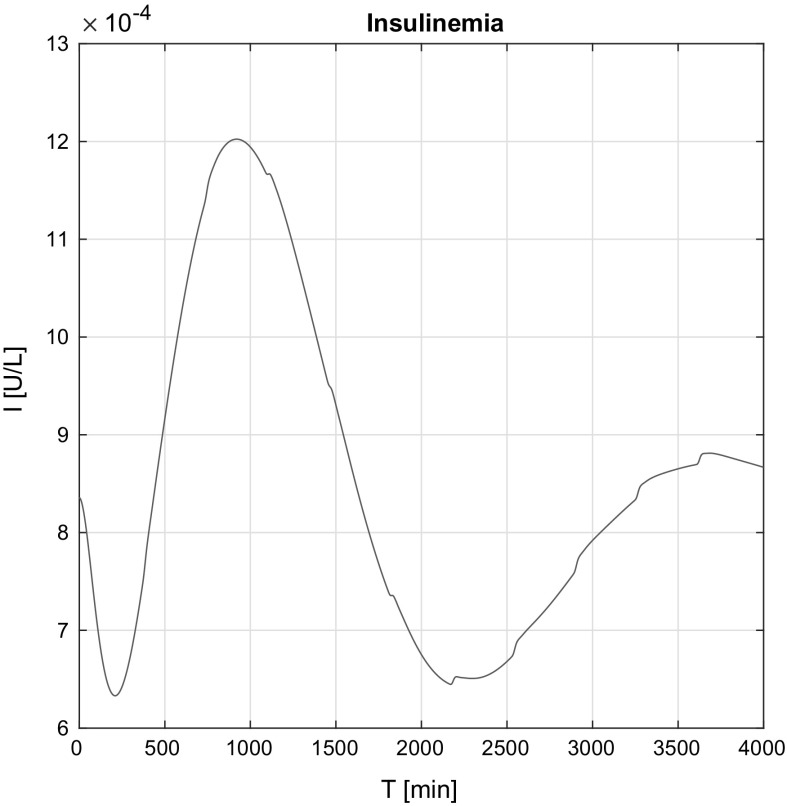
Simulation result of insulinemia *I*(*t*) in the affine case

Figure 12 shows insulinemia variation *I*(*t*) over time. It can be seen that the control insulin signal results a stable internal insulin level, nonetheless the presence of the insulin peaks. The delays of the effect coming from the model’s structure are also visible, since despite the immediate insulin signal from the beginning the insulinemia initially decays, but it stabilizes over time.

**Fig. 13:**
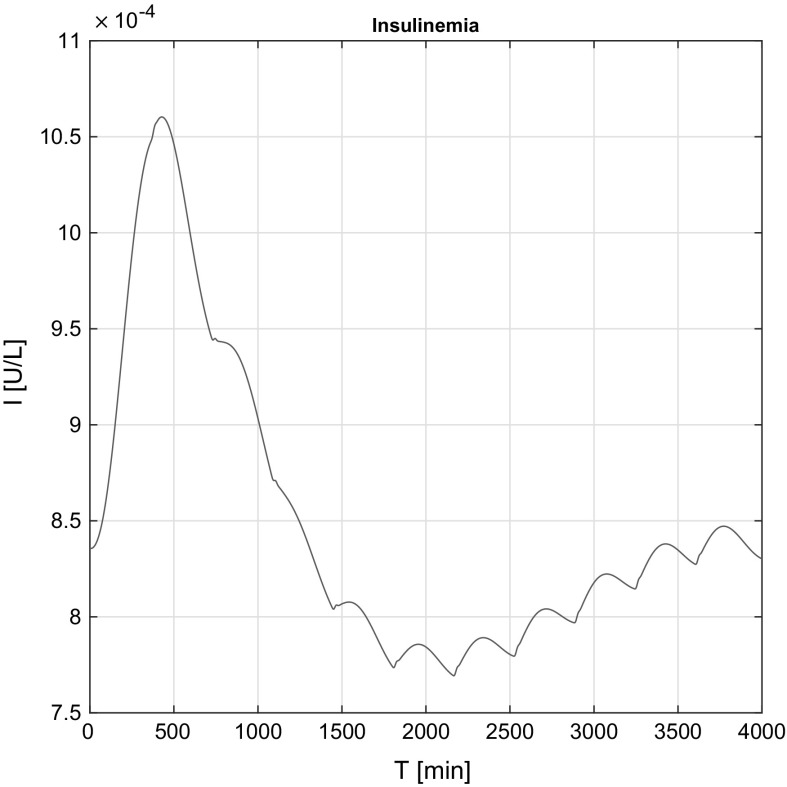
Simulation result of insulinemia *I*(*t*) in the non-affine case

In the non-affine case (Fig. 13), the waviness appears come from the insulin peaks.

## Conclusion

In this paper, the usability of RFPT-based controller design method was reported in the case of a type 1 diabetes nonlinear model optimized to long-term identification purposes, but used for control purposes as well. In line with the requirements of the T1DM model, an appropriate feed intake function was designed and long simulation time was used in order to provide unfavorable circumstances to test the behavior of the developed RFPT-based controller. During this study, fixed control parameters were used without advanced optimization techniques. On these considerations, the applicability of the novel RFPT method has been demonstrated. With online optimization techniques, better performance is expected that is a next step of the presented research.
